# Overexpression of melanocortin 2 receptor accessory protein 2 (MRAP2) in adult paraventricular MC4R neurons regulates energy intake and expenditure

**DOI:** 10.1016/j.molmet.2018.09.010

**Published:** 2018-10-04

**Authors:** Giuseppe Bruschetta, Jung Dae Kim, Sabrina Diano, Li F. Chan

**Affiliations:** 1 Program in Integrative Cell Signaling and Neurobiology of Metabolism, Yale University School of Medicine, New Haven, CT, 06520, USA; 2 Department of Obstetrics, Gynecology, and Reproductive Sciences, Yale University School of Medicine, New Haven, CT, 06520, USA; 3 Department of Neuroscience, Yale University School of Medicine, New Haven, CT, 06520, USA; 4 Department of Comparative Medicine, Yale University School of Medicine, New Haven, CT, 06520, USA; 5 Department of Clinical Medicine and Surgery, University of Naples “Federico II”, Naples, Italy; 6 Centre for Endocrinology, William Harvey Research Institute, Barts and the London School of Medicine, Queen Mary University of London, Charterhouse Square, London EC1M 6BQ, UK

**Keywords:** MRAP2, MC4R, Food intake, Energy homeostasis, Obesity

## Abstract

**Objective:**

Melanocortin 2 receptor accessory protein 2 (MRAP2) has a critical role in energy homeostasis. Although MRAP2 has been shown to regulates a number of GPCRs involved in metabolism, the key neurons responsible for the phenotype of gross obesity in MRAP2 deficient animals are unclear. Furthermore, to date, all the murine MRAP2 models involve the prenatal deletion of MRAP2.

**Methods:**

To target Melanocortin 4 receptor (MC4R)-expressing neurons in the hypothalamic paraventricular nucleus (PVN), we performed stereotaxic surgery using AAV to selectively overexpress MRAP2 postnatally in adult *Mc4r-cre* mice. We assessed energy homeostasis, glucose metabolism, core body temperature, and response to MC3R/MC4R agonist MTII.

**Results:**

*Mc4r-cre*^*PVN-MRAP2*^ female mice on a standard chow diet had less age-related weight gain and improved glucose/insulin profile compared to control *Mc4r-cre*^*PVN-GFP*^ mice. These changes were associated with a reduction in food intake and increased energy expenditure. In contrast, *Mc4r-cre*^*PVN-MRAP2*^ male mice showed no improvement on a chow diet, but improvement of energy and glucose metabolism was observed following high fat diet (HFD) feeding. In addition, an increase in core body temperature was found in both females fed on standard chow diet and males fed on HFD. *Mc4r-cre*^*PVN-MRAP2*^ female and male mice showed increased neuronal activation in the PVN compared to controls, with further increase in neuronal activation post MTII treatment in females.

**Conclusions:**

Our data indicate a site-specific role for MRAP2 in PVN MC4R-expressing neurons in potentiating MC4R neuronal activation at baseline conditions in the regulation of food intake and energy expenditure.

## Highlights

• Postnatal overexpression of MRAP2 regulates energy balance, thermogenesis and glucose metabolism.
• Overexpression of MRAP2 in MC4R expressing neurons increases PVN neuronal activation.
• There is a sex difference in extent of metabolic protection, with a more marked lean phenotype in females.

## 1 Introduction

MRAP2 is predominantly expressed in the hypothalamus, in particular within the paraventricular nucleus (PVN), a region known to express MC4R and with a critical role in energy homeostasis [1], [2], [3]. Mice with global MRAP2 deletion and conditional MRAP2 deletion in SIM1 expressing neurons developed marked obesity, while rare loss-of-function or missense heterozygous variants in MRAP2 were identified in humans with severe early-onset obesity [4], [5]. MRAP2's critical role in the control of energy homeostasis has been linked to action on MC4R signaling [4], [6]. Further evidence that MRAP2 acts via MC4R signaling came from a study on the role of Mrap2 in zebrafish feeding and growth [7].

Although data point to an MC4R dependent function, *Mrap2*^−/−^ mice do not fully phenocopy the *Mc4r*^−/−^ mice. In particular, there is the paradox that while *Mrap2*^−/−^ mice become obese without detectable changes in food intake or energy balance, *Mc4r*^−/−^ mice have hyperphagia and reduced energy expenditure (EE) [8], [9]. *Mrap2*^−/−^ mice remain responsive to treatment with MTII, a MC3R/MC4R agonist, while the anorexic response to MTII is abolished in *Mc4r*^−/−^ mice, suggesting at least some preservation of MCR function centrally [4], [10].

The phenotype of gross, early onset obesity without detectable change of food intake and energy expenditure, replicated in an independent Mrap2 deficient model, is particularly intriguing [4], [6]. However, the mechanism by which MRAP2 knockout animals become obese is still unclear. Plasma corticosterone, thyroid function, faecal energy measurements, body temperature, and brown fat function in response to cold challenge were all indistinguishable between *Mrap2*^−/−^ and *Mrap2*^+/+^ mice [4], [6]. Some of the complexity may arise from the fact that MRAP2 is a promiscuous accessory protein [1]. In addition to the melanocortin receptor family (MCR) [11], MRAP2 interacts and regulates other G Protein-Coupled Receptors (GPCRs) beyond the MCR family [1], [12], [13], [14]. Interaction with Prokineticin 1 receptor, orexin 1 receptor, and Ghrelin receptor have been reported [12], [13], [14]. However, these interactions would result in a lean phenotype in the absence of MRAP2. Thus, while interaction with these additional GPCRs forms part of the growing understanding of MRAP2 action in the neuronal control of energy homeostasis, it does not explain the obese phenotype of MRAP2 deficient animals.

With data pointing to the PVN and MC4R as the key to unraveling the obesity phenotype in MRAP2 deficient mice and because all existing mouse models to date involve developmental deletion of MRAP2, we undertook this study to assess the effect of postnatal overexpression of MRAP2 in MC4R neurons of the PVN. Furthermore, using this methodology we are able to exclude the effects of MRAP2 interaction with non-PVN MC4R GPCRs, in particular those GPCRs that have been described to interact with MRAP2 in the arcuate nucleus [12], [13], [14].

## 2 Material and methods

### 2.1 Ethics and animal husbandry

All animal studies on male and female mice were approved by Yale University Institutional Animal Care and Use Committee. Animals had free access to water and food was provided *ad libitum*, unless otherwise stated. Mice were fed on a standard chow diet (SD) (diet no. 2018; 18% calories from fat; Teklad Diets, Harlan Laboratories) for up to 6 months of age. For high fat diet (HFD) experiments, male mice were exposed to HFD (category no. 93075; 45% fat; Teklad Diets, Harlan Laboratories) starting at 2 weeks after PVN injection. All animals were kept in temperature and humidity-controlled rooms, in a 12 h dark and 12 h light cycle, with lights on from 7:00 AM to 7:00 PM.

### 2.2 Generation of *Mc4r-t2a-cre* mice and genotyping

*Mc4r-t2a-cre* mice were generated as previously described [15]. Genomic DNA was isolated from tails or yolk sacs by standard methods. *Mc4r-t2a-cre* mice were genotyped by polymerase chain reaction (PCR parameters: 42 cycles, 93 °C for 30 s, 56 °C for 1 min, and 72 °C for 5 min). Amplification of a wild-type (WT) allele generated a 3.6-kb product, and a 4.1-kb product in the case of a mutant allele using the following primers: cre 350 FRT5: 5′-ctgtcacttggtcgtggca-3′, Mc4r-2A-creStop F1 FRT3: 5′-gatcatgtgtaacgccgtc-3, Mc4r-2A-creStop R1 FRT3: 5′-catgtcaattcataacgccc-3.

### 2.3 Stereotaxic viral injection of Adeno-associated virus (AAV) into the PVN

The AAV2-CMV-DIO-GFP [AAV-DIO-GFP] and AAV2-CMV-DIO-MRAP2-FLAG [AAV-DIO-MRAP2] virus (VECTOR BIOLABS) were injected bilaterally into the PVN of Mc4r-cre animals. Moreover AAV2-CMV-GFP (VECTOR BIOLABS cat No. 7004) virus was co-injected to determine successful PVN targeting. Eight weeks old *Mc4r-cre* mice were anesthetized with 100 mg/kg ketamine and 10 mg/kg xylazine (IP) and placed in stereotaxic apparatus. A guide cannula with dummy cannula (Plastics one, Roanoke, VA) was inserted into the PVN according to the atlas of Franklin and Paxinos (Franklin KBJ) (co-ordinates, bregma: anterior-posterior −0.7 mm; lateral ±0.2 mm; and dorsal–ventral −5.2 mm), virus was infused at a rate of 40 nl/min (∼2 × 10^12^ viral particles/mL) for 15 min and the injector (Plastics one) remained in place for an additional 5 min before. The injector was connected with a Hamilton syringe and infusion was administered at a rate of 33.3 nL/min.

### 2.4 GFP immunostaining

Fed mice were perfused and brains were processed for immunofluorescence staining to confirm the injection site in the PVN, using anti-GFP antibody (ab13970; Abcam). Mice in which viral injections were located outside the PVN were studied separately. The sections were incubated overnight in anti-GFP antibody (diluted 1:5000 in 0.1 mol/L sodium phosphate buffer) and then incubated in secondary antibody (category no. A11039, Alexa Fluor 488–coupled goat anti-chicken, 1:500 dilution; Life Technologies) for 2 h. Sections were then analyzed with fluorescent microscope.

### 2.5 FLAG immunostaining

Immunofluorescence staining was performed using anti-FLAG antibody (F1804; Sigma). Brains were sectioned with a vibratome, and sections were incubated for 24 h at room temperature in anti-FLAG antibody (diluted 1:2000). After several washes with phosphate buffer (PB), sections were incubated in secondary antibody (category no. BA 2000 biotinylated horse anti-mouse IgG; 1:200 in PB; Vector Laboratories) for 2 h at room temperature, then rinsed in PB three times 10 min each time, and incubated for 2 h at room temperature with Alexa Fluor 594 streptavidin (Life Technologies, 1:2000 in PB). Sections were mounted on glass slide with vectashield (Vector lab) and analyzed with fluorescent microscope.

### 2.6 Indirect calorimetry system and body composition

Body weight was measured weekly after stereotaxic surgery. Body composition was measured *in vivo* by MRI (EchoMRI; Echo Medical Systems, Houston, TX). Twelve weeks after PVN injection male and female mice were acclimated in metabolic chambers (TSE Systems, Germany) for 4 days before the start of the recordings. Animals were continuously recorded for 3 days with the following measurements being taken every 30 min. Measurements include food intake, locomotor activity (in X and Y axes), and gas exchange (O_2_ and CO_2_) every 30 min using the TSE LabMaster System. Respiratory exchange ratio (RER) was calculated as a ratio of CO_2_ production and O_2_ consumption. Energy expenditure (EE) was calculated according to the manufacturer's guidelines (PhenoMaster Software, TSE Systems) and analyzed relative to body weight using ANCOVA analysis [16]. Food intake was determined continuously using weighing sensors integrated within the sealed cage.

### 2.7 Glucose and insulin tolerance tests

Glucose tolerance test (GTT) was performed using 2 mg/kg glucose in saline (DeltaSelect) given intraperitoneally (IP) to 16 h fasted animals as previously described [17]. Blood glucose levels were then monitored at 15, 30, 60, and 120 min from the injection. Insulin tolerance test (ITT) was performed using an insulin dose of 0.75 U/kg (Actrapid; Novo Nordisk A/S Denmark) delivered by IP in mice fed *ad libitum*. Blood glucose was measured before IP injection and at 15, 30, 60, and 120 min after insulin injection.

### 2.8 cfos immunostaining

*Ad libitum* fed mice were anesthetized and transcardially perfused with 0.9% saline with heparin followed by 4% paraformaldehyde. In another set of experiments, mice were injected with either MTII (200 nM, IP) or equal volume saline. Animals were perfused 1 h post injection. Brains were collected and post-fixed overnight before several sections of the entire hypothalamus were taken at every 50 μm. Sections were washed and incubated with the goat anti-cfos antibody (Santacruz, 1:2000), and rabbit anti-POMC antibody (Phoenix Pharmaceuticals, 1:2000) in PB containing 4% normal donkey serum, and 1% Triton X-100 for 24 h at room temperature. After several washes with phosphate buffer (PB), sections were incubated in the secondary antibodies (biotinylated donkey anti-goat immunoglobulin G [IgG]; 1:200 in PB; Vector Laboratories and donkey anti-rabbit Alexa-fluor 488; 1:500 in PB; Life Technologies) for 2 h at room temperature, then rinsed in PB five times, 10 min each time. Sections were then mounted on glass slide with VectaShield antifade (Vector Laboratories). Fluorescent images of five to seven brain sections were captured with fluorescent microscope and analyzed by imaging Software (Image J).

### 2.9 Real-time RT PCR

Total RNA from brown adipose tissue was extracted from *Mc4r-cre*^*PVN-MRAP2*^ and control mice using Trizol solution (Invitrogen). Uncoupling protein 1 (UCP1) and Deiodinase 2 (DIO2) mRNA levels in the brown adipose tissue, were measured by real-time PCR. A High Capacity cDNA Reverse transcription Kit (Applied Biosystems) was used for the reverse transcription. Real-time PCR (LightCycler 480; Roche) was performed with diluted cDNAs in a 20-μl reaction volume in triplicates. Primers used for this study are as follows: cat. No. Mm 01244861_m1 for UCP1, cat. No Mm 00515664_m1 for DIO2, and Mm 02619580_g1 for β-actin (Applied Biosystems). The calculations of average Cp values, SDs, and resulting expression ratios for each target gene were based on the Roche LightCycler 480 software.

### 2.10 Statistical analysis

All statistical analysis was performed using GraphPad Prism. Data is plotted as mean ± S.E.M. Student's t test was used to compare two groups; for more than two groups one-way ANOVA was performed followed by Bonferroni multiple comparison test. In all analyses, a two-tailed probability of <5% (i.e., P < 0.05) was considered statistically significant.

## 3 Results

### 3.1 Postnatal overexpression of MRAP2 in PVN MC4R expressing neurons alters metabolism selectively in female *Mc4r-cre*^*PVN-MRAP2*^ mice on a chow diet

**Figure 1:**
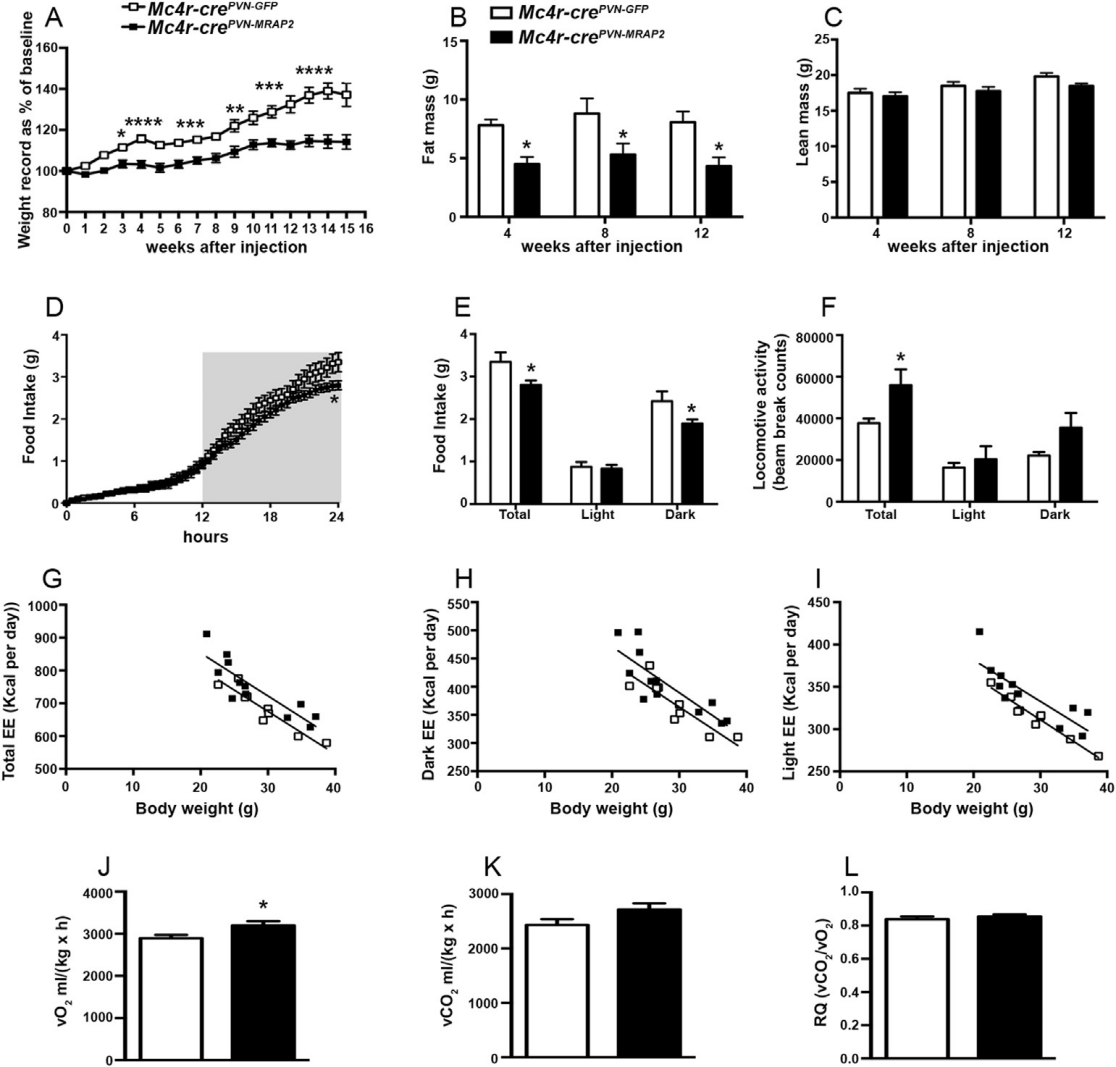
**Selective overexpression of MRAP2 in the PVN MC4R neurons affects metabolism in female mice.** (A–C) Graphs showing body weight (A), fat mass (B), and lean mass (C) of *Mc4r-cre*^*PVN-GFP*^control female mice (n = 11) and *Mc4r-cre*^*PVN-MRAP2*^ (n = 11) female mice at 4, 8 and 12 weeks after injection. (D and E) Graphs showing food intake in *Mc4r-cre*^*PVN-GFP*^ female mice (n = 8) and *Mc4r-cre*^*PVN-MRAP2*^ female mice (n = 10). Total cumulative food intake in the 24 h cycle is shown (D) and in the dark and light phases of the cycle (E). Gray area in D represents the dark phase. (F–I) Graphs showing locomotor activity (F) and total energy expenditure in the 24-h period (G) and in the dark (H) and light (I) phases of the cycle of *Mc4r-cre*^*PVN-GFP*^ (n = 8) and *Mc4r-cre*^*PVN-MRAP2*^ mice (n = 10) three months after the PVN viral injections. (J–L) Graphs showing O_2_ consumed (J), CO_2_ produced (K), and the ratio in (L) of *Mc4r-cre*^*PVN-GFP*^ (n = 8) and *Mc4r-cre*^*PVN-MRAP2*^ mice (n = 10) three months after the PVN viral injections. All data are presented as mean ± SEM. *P < 0.05; **P < 0.01; ***P < 0.001 compared to *Mc4r-cre*^*PVN-GFP*^ mice.

**Figure 2:**
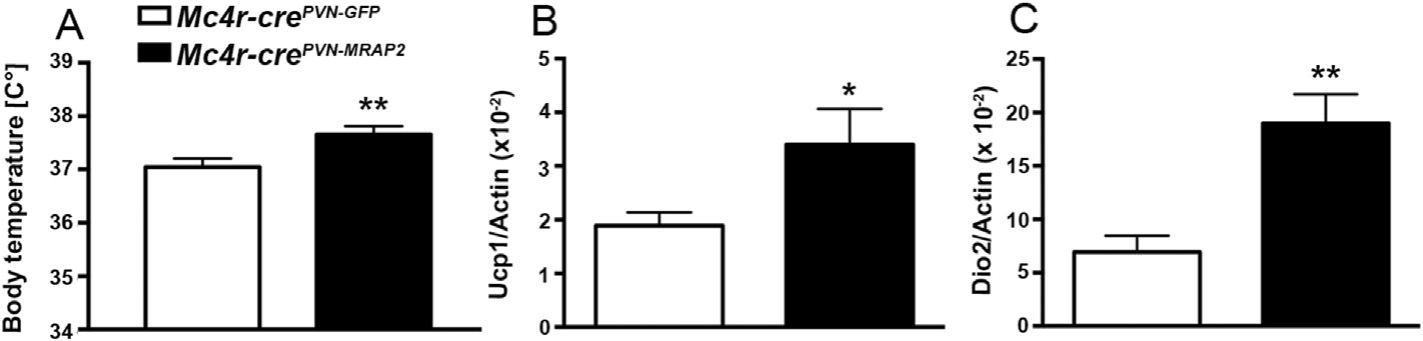
**Effects of PVN-MRAP2 overexpression in MC4R expressing neurons on body temperature and brown adipose tissue of female mice.** (A) Graph showing body temperature in *Mc4r-cre*^*PVN-GFP*^ female mice (n = 7) and *Mc4r-cre*^*PVN-MRAP2*^ female mice (n = 7) three months after PVN viral injections. (B and C) Graphs showing Real-Time RT PCR data for *Ucp1* (B) and *Dio2* (C) in brown adipose tissue of female *Mc4r-cre*^*PVN-GFP*^ (n = 7) and *Mc4r-cre*^*PVN-MRAP2*^ mice (n = 7) three months after PVN viral injections. All data are presented as mean ± SEM. *P < 0.05; **P < 0.01 compared to *Mc4r-cre*^*PVN-GFP*^ mice.

**Figure S1:**
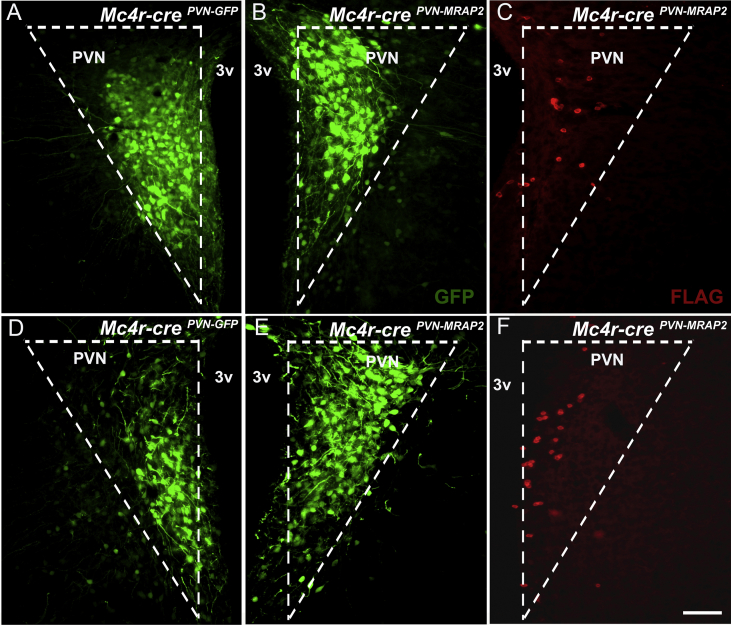
Injection of Adeno-associated virus into the PVN of *Mc4r-cre* mice. (A and B) Representative micrographs of the injection site of non-cre dependent AAV2-CMV-GFP in *Mc4r-cre*^*PVN-GFP*^ (A) and *Mc4r-cre*^*PVN-MRAP2*^ (B) female mice. (C) Representative hypothalamic sections from a female *Mc4r-cre*^*PVN-MRAP2*^ mouse immunostained for FLAG (red) in the hypothalamic paraventricular nucleus (PVN). (D and E) Representative micrographs of the injection site of non-cre dependent AAV2-CMV-GFP in male (D) *Mc4r-cre*^*PVN-GFP*^ and (E) *Mc4r-cre*^*PVN-MRAP2*^. (F) Representative hypothalamic sections from a male *Mc4r-cre*^*PVN-MRAP2*^ mouse immunostained for FLAG (red) in the hypothalamic paraventricular nucleus (PVN). 3v = third ventricle; PVN = paraventricular nucleus of the hypothalamus. Bar scale in F (for A–F) represents 100 μm.

**Figure S2:**
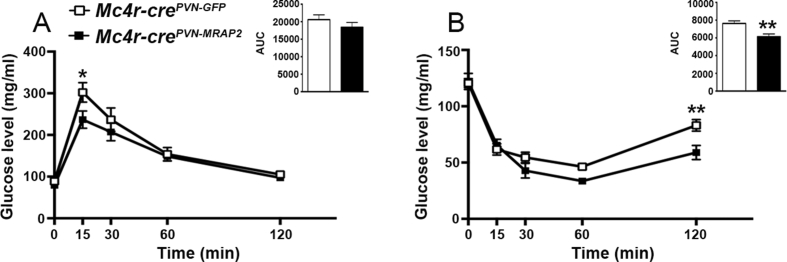
MRAP2 overexpression in PVN MC4R neurons improves glucose metabolism in female mice. (A) Graph showing glucose tolerance test in female *Mc4r-cre*^*PVN-GFP*^ and *Mc4r-cre*^*PVN-MRAP2*^ mice after 3 months for the PVN viral injections (n = 11 per group). (B) Graph showing insulin tolerance test in female *Mc4r-cre*^*PVN-GFP*^ and *Mc4r-cre*^*PVN-MRAP2*^ mice after 3 months for the PVN viral injections (n = 11 per group). All data are presented as mean ± SEM. *P < 0.05, **P < 0.01 compared to control mice.

**Figure S3:**
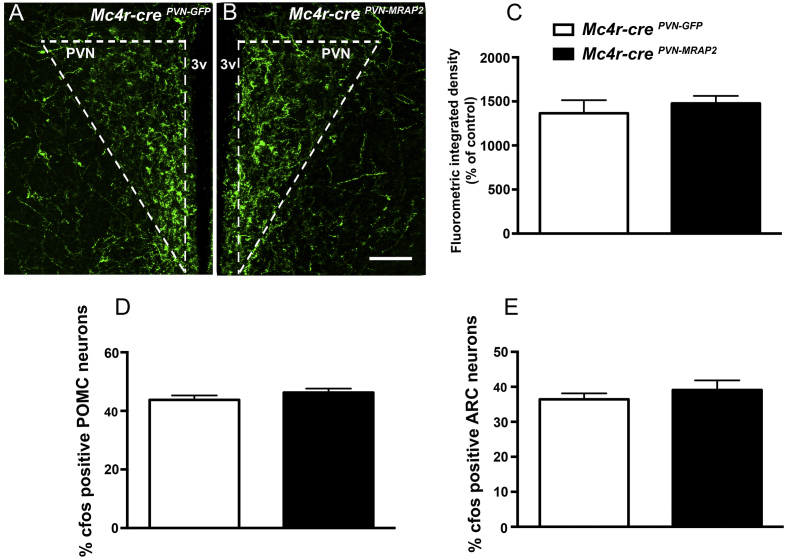
MRAP2 overexpression in PVN MC4R neurons does not alter POMC staining or activation in female mice. (A and B) Representative light micrographs showing POMC immunostained fibers in the PVN of a female *Mc4r-cre*^*PVN-GFP*^ mouse (A) and a female *Mc4r-cre*^*PVN-MRAP2*^ mouse (B). (C) Graph showing the integrated density quantification of POMC fibers in the PVN of female *Mc4r-cre*^*PVN-GFP*^ and *Mc4r-cre*^*PVN-MRAP2*^ mice (n = 6 per group). (D) Quantification of cfos expression in POMC neurons of female *Mc4r-cre*^*PVN-GFP*^ and *Mc4r-cre*^*PVN-MRAP2*^ mice (n = 6 per group). (E) Graph showing the quantification of cfos expression in the arcuate nucleus of hypothalamus of *Mc4r-cre*^*PVN-GFP*^ and *Mc4r-cre*^*PVN-MRAP2*^ mice (n = 6 per group). All data are presented as mean ± SEM. Bar scale in B (for A and B) represents 100 μm.

To assess the role of MRAP2 in the MC4R-expressing neurons of the PVN postnatally, we injected an *AAV-DIO-MRAP2* and its control, *AAV-DIO-GFP*, in the PVN of female (Figure S1A–B) and male (Figure S1D–E) *Mc4r-cre* mice [15]. Moreover, to assess the overexpression of MRAP2 in the PVN, immunostaining for FLAG epitope was performed in female (Figure S1C) and in male *Mc4r-cre*^*PVN-MRAP2*^ mice (Figure S1F). To determine whether selective MRAP2 overexpression in PVN MC4R affects metabolism, metabolic analyses were performed in both male and female mice. *Mc4r-cre*^*PVN-MRAP2*^ female mice fed on a standard chow diet showed lower body weight (n = 11 per group; Figure 1A) starting at 3 weeks from the viral injections compared with *Mc4r-cre*^*PVN-GFP*^ controls. The lower body weight was due to a significant reduction of fat mass (4.49 ± 0.61 g n = 11; Figure 1B) in *Mc4r-cre*^*PVN-MRAP2*^ female mice compared to female controls (7.80 ± 0.51 g *Mc4r-cre*^*PVN-GFP*^ female mice; n = 11) evidenced after 4 weeks post viral injections. No differences in lean mass were observed between the 2 experimental groups (n = 11; Figure 1C). This was associated with decreased food intake (2.79 ± 0.10 g in female *Mc4r-cre*^*PVN-MRAP2*^ mice and 3.35 ± 0.22 g in female *Mc4r-cre*^*PVN-GFP*^ controls; Figure 1D–E) specifically during the dark period (2.41 ± 0.23 g in controls vs 1.89 ± 0.10 g in *Mc4r-cre*^*PVN-MRAP2*^ female mice; n = 8 and 11, respectively; Figure 1D–E). Furthermore, increases in locomotor activity (37,744 ± 2138 beam break counts in controls vs 55,914 ± 7631 beam break counts in *Mc4r-cre*^*PVN-MRAP2*^ female mice; n = 8 and 10, respectively; Figure 1F) and energy expenditure (Figure 1G–I) were also observed in *Mc4r-cre*^*PVN-MRAP2*^ female mice compared to female controls. vO_2_ was also different between *Mc4r-cre*^*PVN-MRAP2*^ female mice (3195.63 ± 101.02 ml per day) and controls (2890.92 ± 80.74 ml per day, Figure 1J), while no differences in vCO_2_ (2430.37 ± 104.96 ml per day in controls vs 2715.73 ± 113.60 ml per day in *Mc4r-cre*^*PVN-MRAP2*^ mice) and in respiratory quotient were observed (0.83 ± 0.01 ml per day in controls vs 0.85 ± 0.01 ml per day in *Mc4r-cre*^*PVN-MRAP2*^ mice; Figure 1K–L) between the experimental groups. In agreement with the improved metabolic phenotype, both glucose (Figure S2A) and insulin tolerance tests (Figure S2B) were significantly improved in *Mc4r-cre*^*PVN-MRAP2*^ female mice compared to female *Mc4r-cre*^*PVN-GFP*^ controls. Arcuate POMC cells are known to activate MC4R-expressing neurons in the PVN, inhibiting food intake and increasing energy expenditure. To ensure that the observed improved metabolic phenotype was not due to a differential activation of POMC neurons, we quantified PVN POMC fiber density (1366.35 ± 147.24 in controls vs 1478.14 ± 83.66 in *Mc4r-cre*^*PVN-MRAP2*^ n = 6; Figure S3A–C) and arcuate nucleus neuronal activation, including POMC (43.78 ± 1.47 in controls vs 46.27 ± 1.36 in *Mc4r-cre*^*PVN-MRAP2*^ n = 6, cfos positive POMC neurons; 36.41 ± 1.74 in controls vs 39.08 ± 2.76 in *Mc4r-cre*^*PVN-MRAP2*^ n = 6 per group, cfos positive cells in the ARC; Figure S3D and E), and found no difference between *Mc4r-cre*^*PVN-MRAP2*^ and *Mc4r-cre*^*PVN-GFP*^ female mice. In addition, a significant increase in body temperature was also found in *Mc4r-cre*^*PVN-MRAP2*^ female mice (37.65 ± 0.15 °C) compared to *Mc4r-cre*^*PVN-GFP*^ female mice (37.04 ± 0.16 °C; Figure 2A) that was associated with significant increases in UCP1 (Figure 2B) and Dio2 (Figure 2C) mRNA levels in the BAT. Altogether, these data indicate that MRAP2 in PVN MC4R neurons affects energy metabolism in female mice.

### 3.2 Selective MRAP2 overexpression in PVN MC4R neurons induced increased PVN neuronal activation, which is further affected by MTII administration, in female mice

**Figure 3:**
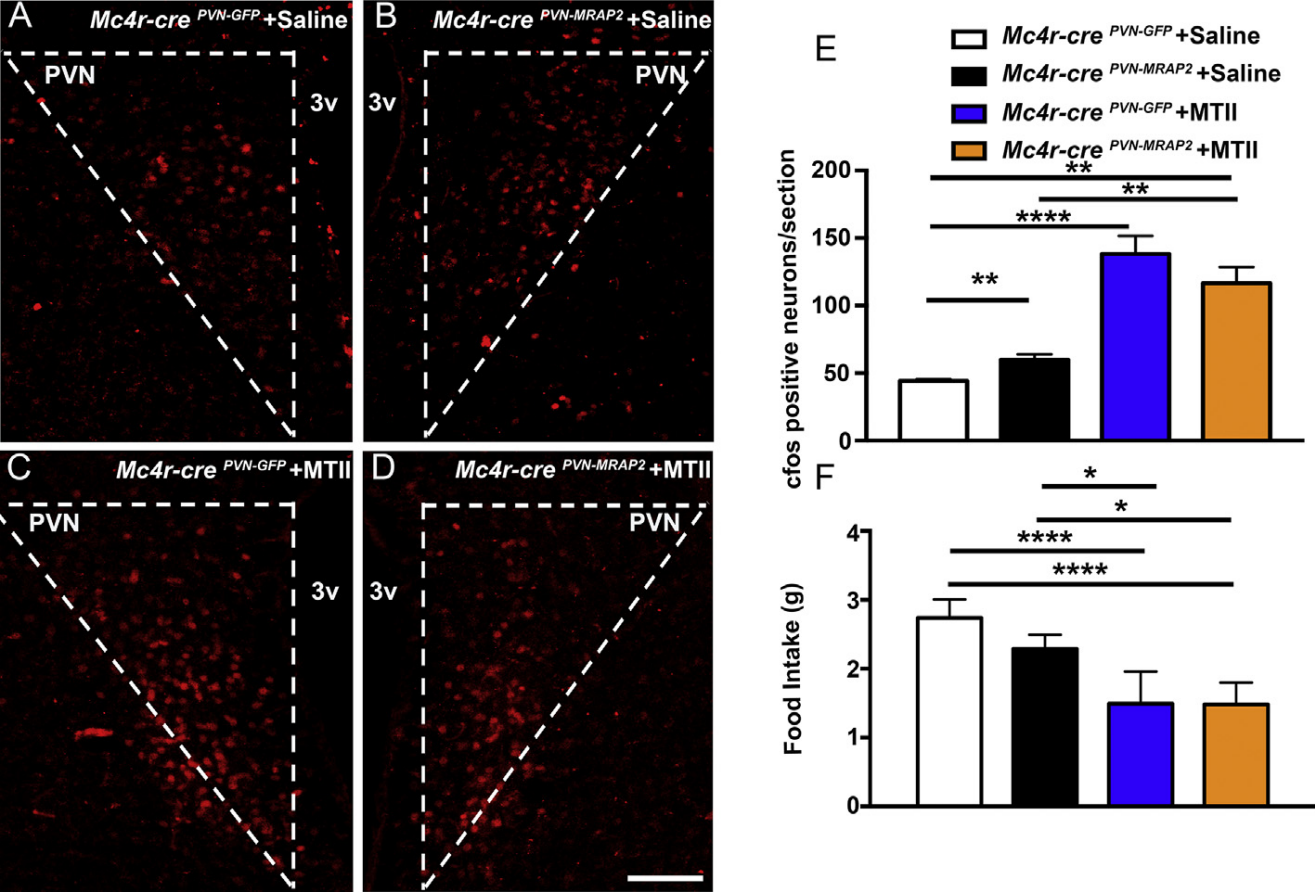
**Selective overexpression of MRAP2 in MC4R expressing neurons in the PVN affects neuronal activation in female mice which is further increased with MTII administration.** (A and B) Representative hypothalamic sections from a female *Mc4r-cre*^*PVN-GFP*^ (A) and a female *Mc4r-cre*^*PVN-MRAP2*^ mouse both injected IP with saline (B) immunostained for cfos (red) in the hypothalamic paraventricular nucleus (PVN). (C and D) Representative hypothalamic PVN sections immunostained for cfos from a *Mc4r-cre*^*PVN-GFP*^ (C) and a *Mc4r-cre*^*PVN-MRAP2*^ mouse (D) following IP injection with MTII. (E) Quantification of cfos expression in the PVN of *Mc4r-cre*^*PVN-GFP*^ (n = 5) and *Mc4r-cre*^*PVN-MRAP2*^ female mice (n = 6) post IP saline and cfos expression in the PVN of *Mc4r-cre*^*PVN-GFP*^ (n = 4) and *Mc4r-cre*^*PVN-MRAP2*^ female mice (n = 4) injected with MTII. (F) Graph showing no differences in feeding responses (food intake over 24 h) after peripheral injection of MTII in female *Mc4r-cre*^*PVN-GFP*^ (n = 4) and *Mc4r-cre*^*PVN-MRAP2*^ mice (n = 4) three months after the PVN viral injections. A significant difference is noted between saline treated and MTII treated animals in both groups. 3v = third ventricle; PVN = paraventricular nucleus of the hypothalamus. Bar scale in A–D represents 100 μm. All data are presented as mean ± SEM. **P < 0.01 compared to *Mc4r-cre*^*PVN-GFP*^ mice.

As MRAP2 overexpression in PVN-MC4R neurons in adult female mice led to a leaner phenotype, to determine the effect of MRAP2 overexpression on PVN neuronal activation, we performed and quantified cfos immunostaining in the PVN of *Mc4r-cre*^*PVN-MRAP2*^ and *Mc4r-cre*^*PVN-GFP*^ female mice. A significant increase of cfos immunoreactivity was found in *Mc4r-cre*^*PVN-MRAP2*^ female mice (59.97 ± 3.88 cfos positive cells/section; n = 6 Figure 3B, E) compared to *Mc4r-cre*^*PVN-GFP*^ female mice (44.20 ± 1.39 cfos positive cells/section; n = 5 Figure 3A, E).

Furthermore, when MTII, a MC3R/MC4R agonist, was peripherally injected, no difference in food intake (Figure 3F) and cfos activation in the PVN (Figure 3C–E) was observed between *Mc4r-cre*^*PVN-GFP*^ and *Mc4r-cre*^*PVN-MRAP2*^ female mice (1.49 ± 0.46 g and 138.24 ± 13.35 cfos positive cells/section in controls vs 1.48 ± 0.31 g and 116.54 ± 12.08 cfos positive cells/section in *Mc4r-cre*^*PVN-MRAP2*^ female mice; n = 4 per group; Figure 3E). Comparing saline and MTII treated *Mc4r-cre*^*PVN-MRAP2*^ female mice, a statistical difference was noted in food intake and cfos positive cells (59.97 ± 3.88 cfos positive cells/section; n = 6 compared with 116.54 ± 12.08 cfos positive cells/section in *Mc4r-cre*^*PVN-MRAP2*^ female mice; n = 4 per group Figure 3E,F).

### 3.3 Selective overexpression of MRAP2 in MC4R neurons does not alter energy metabolism in male mice on a standard chow diet

**Figure 4:**
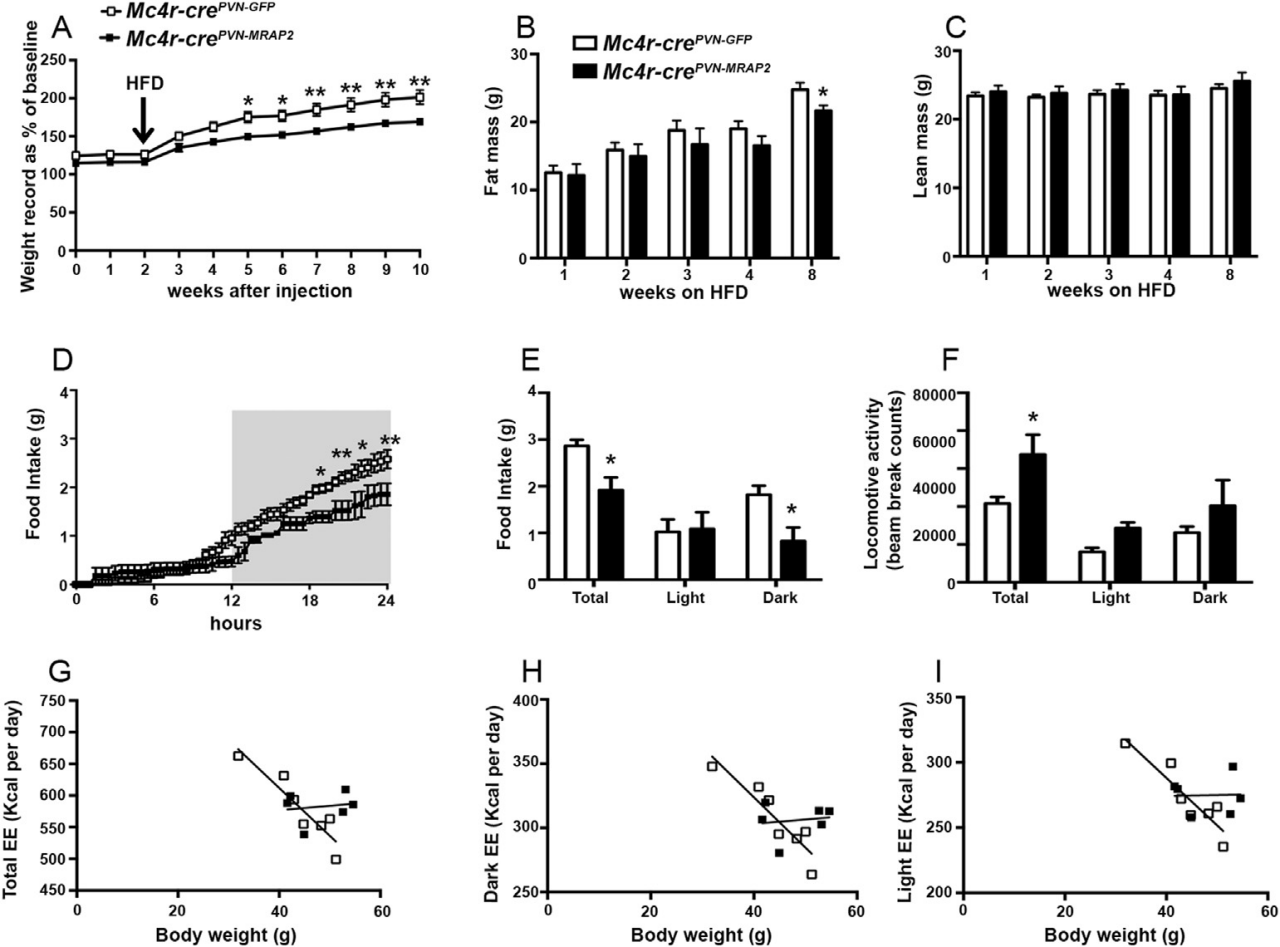
**Selective overexpression of MRAP2 in PVN MC4R expressing neurons affects metabolism in male mice on HFD.** (A–C) Graphs showing body weight (A), fat mass (B) and lean mass (C) of male *Mc4r-cre*^*PVN-GFP*^ (*n* = 6) and *Mc4r-cre*^*PVN-MRAP2*^ mice (*n* = 6) exposed to HFD at 2 weeks after PVN viral injections. (D and E) Graphs showing food intake in male *Mc4r-cre*^*PVN-GFP*^ (*n* = 7), and *Mc4r-cre*^*PVN-MRAP2*^ mice (*n* = 7) at 8 weeks of HFD. Cumulative food intake over a 24 h period, feeding during light and dark phases are shown. Gray area represents the dark phase. (F–I) Graphs showing locomotor activity (F), energy expenditure as a total in the 24-h cycle (G) and in the dark (H) and light (I) phases of the cycle of *Mc4r-cre*^*PVN-GFP*^ (n = 7) and *Mc4r-cre*^*PVN-MRAP2*^ mice (n = 7) at 8 weeks of HFD. All data are presented as mean ± SEM. *P < 0.05; **P < 0.01 compared to *Mc4r-cre*^*PVN-GFP*^ mice.

**Figure 5:**
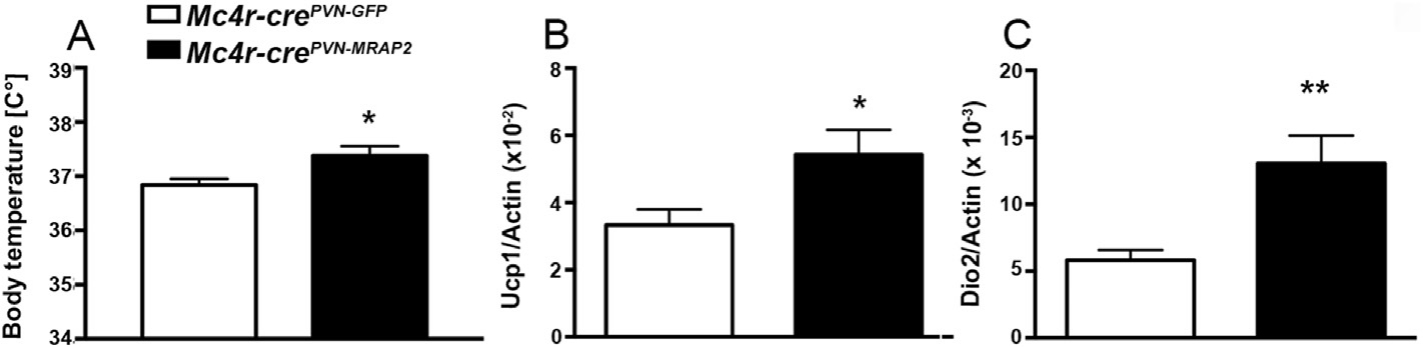
**Effects of PVN-MRAP2 overexpression on body temperature and brown adipose tissue of male mice on HFD.** (A) Graph showing body temperature in male *Mc4r-cre*^*PVN-GFP*^ (n = 7) and *Mc4r-cre*^*PVN-MRAP2*^ mice (n = 7). (B and C) Graphs showing Real-Time RT PCR data for *Ucp1* (B) and *Dio2* (C) in the brown adipose tissue of male *Mc4r-cre*^*PVN-GFP*^ (n = 7) and *Mc4r-cre*^*PVN-MRAP2*^ mice (n = 7) after 8 weeks of HFD. All data are presented as mean ± SEM. *P < 0.05 compared to *Mc4r-cre*^*PVN-GFP*^ mice; **P < 0.01 compared to *Mc4r-cre*^*PVN-GFP*^ mice.

**Figure S4:**
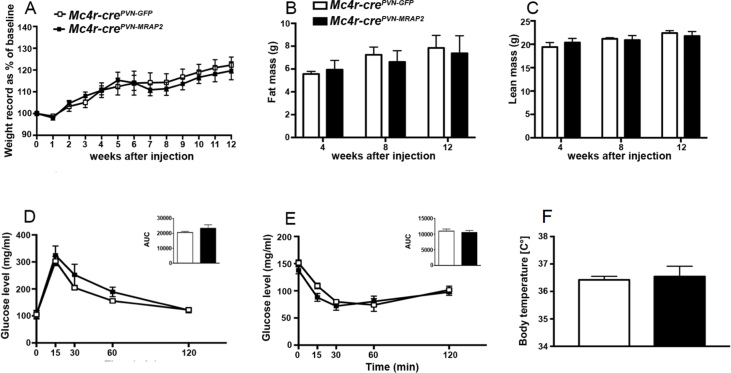
MRAP2 overexpression in the PVN does not affect metabolism of male mice exposed to a standard chow diet. (A–C) Graphs showing body weight (A), fat mass (B), and lean mass (C) of male *Mc4r-cre*^*PVN-GFP*^control (n = 6) and *Mc4r-cre*^*PVN-MRAP2*^ (n = 6) mice at 4, 8 and 12 weeks after PVN viral injections. (D and E) Graphs showing glucose tolerance (D) and insulin tolerance tests in male *Mc4r-cre*^*PVN-GFP*^ and *Mc4r-cre*^*PVN-MRAP2*^ mice (n = 6 per group) after 3 months from the PVN viral injections. (F) and rectal body temperature in male *Mc4r-cre*^*PVN-GFP*^ (n = 7) and *Mc4r-cre*^*PVN-MRAP2*^ mice (n = 6) after 3 months from the PVN viral injections. All data are presented as mean ± SEM.

**Figure S5:**
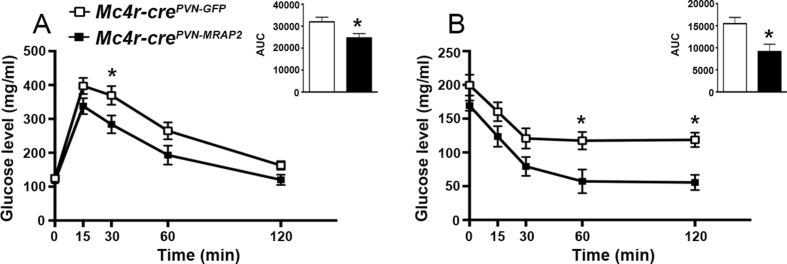
MRAP2 overexpression in the PVN MC4R expressing neurons improves glucose metabolism in male mice exposed to HFD. (A) Graph showing improved glucose tolerance in male *Mc4r-cre*^*PVN-GFP*^ and *Mc4r-cre*^*PVN-MRAP2*^ mice (n = 11 per group) after 8 weeks of HFD feeding. (B) Graph showing insulin tolerance test male *Mc4r-cre*^*PVN-GFP*^ and *Mc4r-cre*^*PVN-MRAP2*^ mice (n = 11 per group) after 8 weeks of HFD feeding. All data are presented as mean ± SEM. *P < 0.05 compared to control mice.

**Figure S6:**
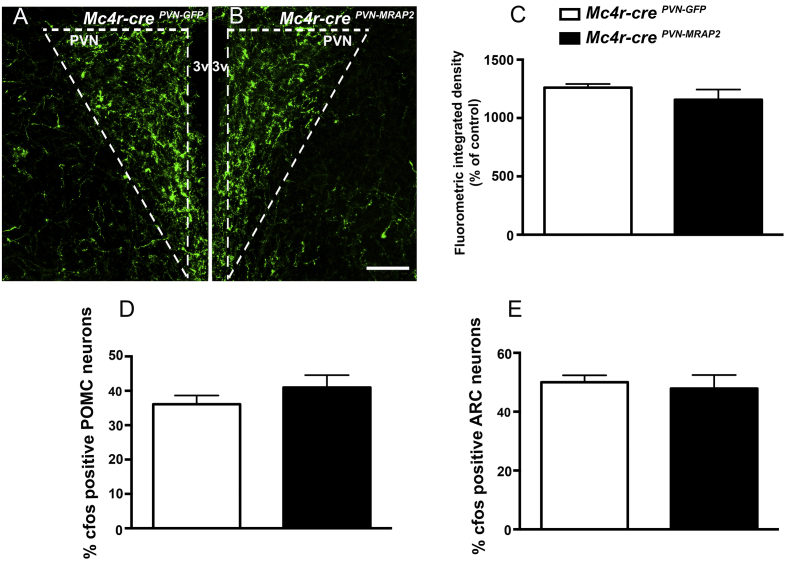
MRAP2 overexpression in the PVN MC4R expressing neurons does not affect POMC staining and activation in male mice exposed to HFD feeding. (A and B) Representative light micrographs showing POMC immunostained fibers in the PVN of a male *Mc4r-cre*^*PVN-GFP*^ (A) and a male *Mc4r-cre*^*PVN-MRAP2*^ mouse (B). (C) Graph showing the integrated density quantification of POMC fibers in the PVN of male *Mc4r-cre*^*PVN-GFP*^ and *Mc4r-cre*^*PVN-MRAP2*^ mice (n = 7 per group) exposed to HFD. (D) Quantification of cfos expression in POMC neurons of *Mc4r-cre*^*PVN-GFP*^ and *Mc4r-cre*^*PVN-MRAP2*^ mice (n = 6 per group) exposed to HFD. (E) Graph showing the quantification of cfos expression in the arcuate nucleus of hypothalamus of male *Mc4r-cre*^*PVN-GFP*^ and *Mc4r-cre*^*PVN-MRAP2*^ mice (n = 6 per group). Bar scale in B (for A and B) represents 100 μm. All data are presented as mean ± SEM.

Due to the clear phenotype in female mice, we then studied male mice and, interestingly, no significant differences in body weight (Figure S4A), fat mass (7.84 ± 1.09 g in controls vs 7.38 ± 1.53 g in *Mc4r-cre*^*PVN-MRAP2*^ n = 6 per group, 12 weeks after injection in the PVN; Figure S4B), and lean mass (22.39 ± 0.50 g in controls vs 21.76 ± 0.94 g in *Mc4r-cre*^*PVN-MRAP2*^ n = 6 per group, 12 weeks after injection in the PVN; Figure S4C) were observed in male mice on a standard chow diet. In addition, no changes in glucose tolerance test (Figure S4D), insulin tolerance test (Figure S4E), and body temperature (36.42 ± 0.13 °C in controls vs 36.54 ± 0.37 °C in *Mc4r-cre*^*PVN-MRAP2*^ n = 6 per group; Figure S4F) were observed between *Mc4r-cre*^*PVN-GFP*^ and *Mc4r-cre*^*PVN-MRAP2*^ male mice exposed to a standard chow diet. However, when exposed to high fat diet (45% of fat), male *Mc4r-cre*^*PVN-MRAP2*^ mice showed lower body weight gain compared to control male *Mc4r-cre*^*PVN-GFP*^ mice (Figure 4A; n = 6 per group). This difference was due to a significant reduction in fat mass (24.75 ± 1.01 g in controls vs 21.66 ± 0.79 g in *Mc4r-cre*^*PVN-MRAP2*^ n = 6 per group, 8 weeks on HFD; Figure 4B). No significant changes in lean mass were found (24.51 ± 0.60 g in controls vs 25.57 ± 1.24 g in *Mc4r-cre*^*PVN-MRAP2*^ n = 6 per group, 8 weeks on HFD; Figure 4C). Food intake analysis showed a significant reduction of feeding in *Mc4r-cre*^*PVN-MRAP2*^ male mice compared to male controls specifically during the dark phase (1.81 ± 0.19 g in controls vs 0.82 ± 0.29 g in *Mc4r-cre*^*PVN-MRAP2*^ n = 6 per group; Figure 4D and E). In addition, locomotor activity (20,778 ± 1747 beam break counts in controls vs 33,736 ± 5222 beam break counts in *Mc4r-cre*^*PVN-MRAP2*^ n = 6 per group; Figure 4F) and energy expenditure (11.699 ± 0.615 kcal per day in controls vs 12.937 ± 0.715 kcal per day in *Mc4r-cre*^*PVN-MRAP2*^ n = 6 per group; Figure 4G–I) analyses also showed significant increases in *Mc4r-cre*^*PVN-MRAP2*^ male mice compared to *Mc4r-cre*^*PVN-GFP*^ male mice. In agreement with an overall improved metabolic phenotype, glucose (Figure S5A) and insulin tolerance (Figure S5B) tests were also improved in *Mc4r-cre*^*PVN-MRAP2*^ male mice compared to *Mc4r-cre*^*PVN-GFP*^ male mice. Finally, similar to females, MRAP2 overexpression in MC4R neurons of the PVN induced a significant increase in body temperature (36.83 ± 0.11 °C in controls vs 37.37 ± 0.17 °C in *Mc4r-cre*^*PVN-MRAP2*^ n = 7 per group; Figure 5A) that was accompanied by greater BAT UCP1 (Figure 5B) and Dio2 (Figure 5C) mRNA levels. Finally, to test that the observed improved metabolic phenotype was not due to a differential activation of POMC neurons, we quantified PVN POMC fiber density (1261.03 ± 31.45 in controls vs 1157.57 ± 86.70 in *Mc4r-cre*^*PVN-MRAP2*^ n = 6 per group; Figure S6A–C) and arcuate nucleus neuronal activation, including POMC (36.11 ± 2.53 in controls vs 40.97 ± 3.59 in *Mc4r-cre*^*PVN-MRAP2*^ n = 6 per group, cfos positive POMC neurons; 50.04 ± 2.32 in controls vs 47.85 ± 4.60 in *Mc4r-cre*^*PVN-MRAP2*^ n = 6 per group, cfos positive cells in the ARC; Figure S6D and E), and found no difference between *Mc4r-cre*^*PVN-MRAP2*^ and *Mc4r-cre*^*PVN-GFP*^ male mice fed on HFD. Altogether these data indicate that overexpression of MRAP2 in MC4R neurons affects energy metabolism in male mice when challenged on HFD feeding.

### 3.4 MTII administration increases PVN cfos activation and decreases food intake of *Mc4r-cre*^*PVN-GFP*^ control male mice but does not affect *Mc4r-cre*^*PVN-MRAP2*^ male mice

**Figure 6:**
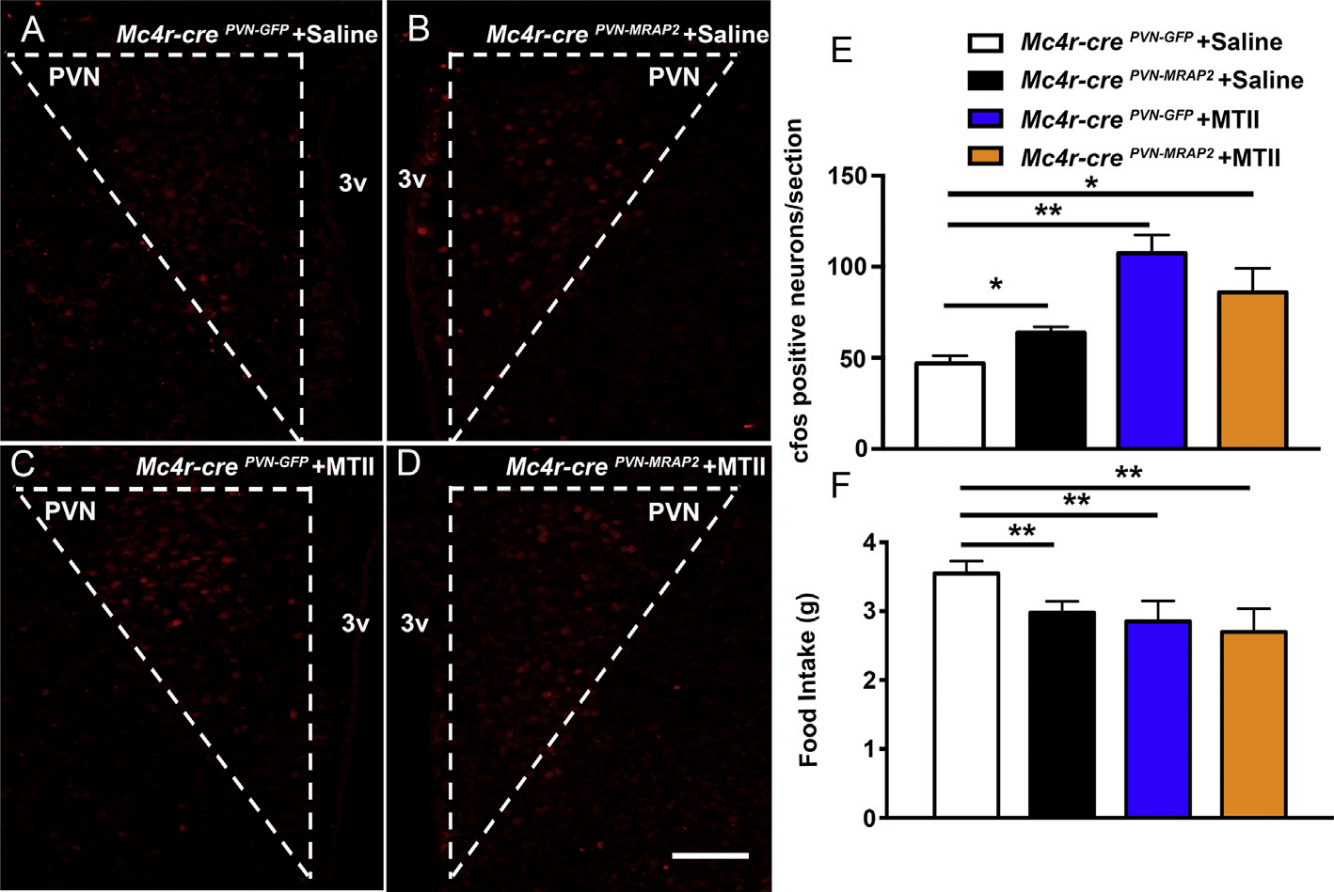
**Effect of peripheral MTII administration on feeding and neuronal activation in *Mc4r-cre*^*PVN-GFP*^ and *Mc4r-cre*^*PVN-MRAP2*^ male mice on HFD.** (A–D) Representative hypothalamic sections immunostained for cfos (red) in male *Mc4r-cre*^*PVN-GFP*^ (A and C) and male *Mc4r-cre*^*PVN-MRAP2*^ mouse (B and D) following IP injection with vehicle (saline; n = 3 per group; A and B) or MTII (n = 6 per group; C and D). (E) Quantification of cfos expression in the PVN of *Mc4r-cre*^*PVN-GFP*^ and *Mc4r-cre*^*PVN-MRAP2*^ male mice injected with saline or MTII. (F) Graph showing feeding response (food intake over 24 h) after peripheral injection of vehicle (saline; n = 3 per group) or MTII (n = 6 per group) in male *Mc4r-cre*^*PVN-GFP*^ and *Mc4r-cre*^*PVN-MRAP2*^ mice on HFD. 3v = third ventricle; PVN = paraventricular nucleus of the hypothalamus. Bar scale in D (for A, B, C, D) represents 100 μm. All data are presented as mean ± SEM. *P < 0.05, **P < 0.01 compared to *Mc4r-cre*^*PVN-GFP*^ control mice.

To assess the response of MTII in *Mc4r-cre*^*PVN-GFP*^ and *Mc4r-cre*^*PVN-MRAP2*^ mice, we assessed the effect of MTII on PVN cfos immunoreactivity and food intake in male mice fed on HFD. Similar to female *Mc4r-cre*^*PVN-MRAP2*^ mice, HFD-fed *Mc4r-cre*^*PVN-MRAP2*^ male mice injected IP with vehicle (saline) showed a significant increase in cfos immunostaining in the PVN compared to vehicle-injected HFD-fed *Mc4r-cre*^*PVN-GFP*^ male mice (46.88 ± 4.34 cfos positive cells in controls vs 63.77 ± 3.22 cfos positive cells in *Mc4r-cre*^*PVN-MRAP2*^ n = 3 per group; Figure 6A, B and E). When MTII was injected IP in both HFD-fed *Mc4r-cre*^*PVN-GFP*^ and *Mc4r-cre*^*PVN-MRAP2*^ male mice, no significant difference was observed (107.37 ± 10.00 cfos positive cells in controls vs 85.97 ± 13.21 cfos positive cells in *Mc4r-cre*^*PVN-MRAP2*^ n = 6 per group; Figure 6C, D and E). In agreement with these data, significantly lower food intake was observed in HFD-fed *Mc4r-cre*^*PVN-MRAP2*^ male mice injected with saline compared to *Mc4r-cre*^*PVN-GFP*^ male mice injected with saline, whilst no difference in food intake was noted between *Mc4r-cre*^*PVN-MRAP2*^ and *Mc4r-cre*^*PVN-GFP*^ male mice injected with MTII (2.97 ± 0.16 g in *Mc4r-cre*^*PVN-MRAP2*^ + saline n = 9, 3.55 ± 0.17 g in *Mc4r-cre*^*PVN-GFP*^ + saline n = 8, 2.70 ± 0.33 g in *Mc4r-cre*^*PVN-MRAP2*^ + MTII n = 6, 2.85 ± 0.29 g in *Mc4r-cre*^*PVN-GFP*^ + MTII n = 5, Figure 6F). Interestingly, unlike in females, no statistical difference in cfos positive cells was observed between *Mc4r-cre*^*PVN-MRAP2*^ male mice treated with saline compared to *Mc4r-cre*^*PVN-MRAP2*^ mice treated with MTII. Together these data suggest a role for MRAP2 in PVN MC4R-expressing neurons in potentiating neuronal activation within the PVN at baseline, and in males without further increase after MTII administration.

## 4 Discussion

MRAP2 has been shown to have a critical role in mammalian metabolism [4]. Mice deficient in MRAP2 have severe early-onset obesity due to increased fat mass. The mechanism of how MRAP2 knockout animals become obese without detectable changes in food intake and energy expenditure remains unclear [1], [3]. Deletion of MRAP2 in Sim-1 expressing neurons leads to obesity in mice to a similar extent as in global *Mrap2*^−/−^ animals pointing to these neurons, located also in the PVN, as key targets of MRAP2 action in metabolism regulation [4]. Although data suggest the involvement of MC4R signaling, the difference in phenotype between the *Mrap2*^−/−^ and *Mc4r*^−/−^ mice suggest possible non-MCR modes of action [4], [6]. This has since been shown to be the case. In addition to MC4R positive neurons, MRAP2 has been shown to have a broader distribution in the Central Nervous System [4], [12], [13], [14], [18]. Within the hypothalamus, MRAP2 has been shown to interact and regulate other GCPRs, including ghrelin receptors expressed in the arcuate Neuropeptide Y/Agouti-related (NPY/AgRP)- expressing neurons, where it positively regulates hunger signaling [13], adding to the complexity of the system.

As Sim1-Cre mice express cre activity in sites outside the PVN [9], [19] and because of the critical role of PVN MC4R in the regulation of metabolism and the changes in neuropeptide transcripts observed in the PVN of global *Mrap2*^−/−^ animals [6], we focused our study on investigating the role of MRAP2 in the MC4R-expressing neurons of the PVN. Our data provide evidence that MRAP2 in MC4R-expressing neurons of the PVN represents an important regulator of food intake and energy metabolism. Furthermore, the data indicate that postnatal manipulation of MRAP2 leads to changes in weight phenotype that are opposite of those observed in *Mrap2*^−/−^ mice in which MRAP2 is deleted during development. By selectively overexpressing MRAP2 in MC4R neurons of the PVN, we reveal a reduction in food intake and energy expenditure that supports the observed lean phenotype, which is unlike that of *Mrap2*^−/−^ in which no change in either measures were identified [4], [6]. This difference could be due to action of MRAP2 on other GPCRs in the arcuate nucleus that leads to lean phenotype in the absence of MRAP2 [12], [13], [14].

In addition, unlike data on the global *Mrap2*-deficient mice that showed no change in BAT activation or body temperature phenotype [4], [6], *Mc4r-cre*^*PVN-MRAP2*^ mice showed a significant increase in BAT activation with increased UCP1 and Dio2 mRNA levels that were associated with increased body temperature compared to control mice in both genders.

We also found a sex-specific metabolic difference in the *Mc4r-cre*^*PVN-MRAP2*^ mice compared with *Mc4r-cre*^*PVN-GFP*^ matched control. Whilst female mice overexpressing MRAP2 in PVN MC4R neurons demonstrate overt protection against obesity on a chow diet, this phenotype was only observed in male mice after challenge with high fat diet. Similarly, sex differences were also observed in locomotor activity and glucose handling. We have previously described a sex-specific increase in daytime locomotor activity and exploratory activity in global *Mrap2*-deficient mice [6]. However, when taken in isolation, focusing on MRAP2-overexpression in PVN MC4R neurons, we now demonstrate an increase in locomotor activity in both female and male *Mc4r-cre*^*PVN-MRAP2*^ mice compared to controls. A difference in glucose clearance and hyperinsulinemia was demonstrated in *Mrap2*^−/−^ mice on a C57/BL6N background, while on a 129/Sv genetic background no changes in insulin and glucose handling were found [4], [6]. Others have confirmed a glucose metabolism phenotype in *Mrap2-*deficient mice on a C57/BL6N background [14]. In agreement with previous work, here we found differences in glucose handling when MRAP2 was manipulated. However, our data suggest that the improved glucose handling was rather a consequence of the leaner phenotype of the female *Mc4r-cre*^*PVN-MRAP2*^ mice. Indeed, no significant differences were observed in male mice on a chow diet.

To determine a possible role for POMC neurons in the regulation of PVN MC4R neurons, we then assessed the activation levels of POMC neurons by cfos immunostaining. No difference in POMC neuronal activation was observed between *Mc4r-cre*^*PVN-MRAP2*^ and controls. Furthermore, no differences were found in POMC fiber staining in the PVN of controls and *Mc4r-cre*^*PVN-MRAP2*^ mice. This suggests that the metabolic phenotype observed might be independent from changes in POMC neuronal activation, thus pointing to the increased neuronal activation in the PVN as the principal cause of the lean phenotype in the *Mc4r-cre*^*PVN-MRAP2*^ model as we observe a significant increase in cfos staining in the PVN of *Mc4r-cre*^*PVN-MRAP2*^ mice compared to controls irrespective of sex.

To assess the hypothesis that MRAP2 overexpression in MC4R neurons affect MC4R signaling, thus affecting food intake, we then administrated MTII, a MC3R/MC4R agonist, in *Mc4r-cre*^*PVN-MRAP2*^ and control mice. MTII induced a significant increase in PVN neuronal activation and a reduction in feeding in male and female *Mc4r-cre*^*PVN-GFP*^ mice. In female *Mc4r-cre*^*PVN-MRAP2*^ mice MTII treatment results in further increase in PVN neuronal activation and decrease in food intake. At baseline, MRAP2 overexpression drives neuronal activation in the PVN in both males and females. However, response to MTII, differed between genders; whilst female *Mc4r-cre*^*PVN-MRAP2*^ mice maintained responsiveness to MTII this was lacking in male *Mc4r-cre*^*PVN-MRAP2*^ mice.

Altogether these results indicate that MRAP2 in PVN MC4R neurons can be manipulated postnatally to result in a lean phenotype in which feeding is reduced and energy expenditure increased along with core body temperature. Importantly, this leads to an increase in neuronal activation with in the PVN. As the number of PVN cfos positive cells are in excess of the number of FLAG positive Mc4r-cre MRAP2 overexpressing cells, the increased cfos would suggest a more global effect on PVN neuronal activation beyond MC4R expressing neurons. The likelihood is that the action of MRAP2 on MC4R PVN neurons is due to the action on enhancing MC4R function as much of the phenotype in *Mc4r-cre*^*PVN-MRAP2*^ mice correlates to models of MC4R activation in which reduced food intake, increased energy expenditure and thermogenesis have been described [9], [15]. Some other features such as sexual dimorphism of responses have not been seen in MC4R activation models. These differences could be due to estrogenic effects on MRAP2/MC4R interaction, which has been described with MRAP1 and MC2R [20], [21], although action on other GPCRs expressed in MC4R expressing PVN neurons cannot be excluded.

## 5 Conclusion

In conclusion, our data provides the first evidence that MRAP2 acts postnatally, in a sex-specific manner, to play a role in the regulation of food intake and energy expenditure through the enhancement of MC4R neuronal signaling in the paraventricular nucleus of hypothalamus.

## Author contribution

GB, JDK, and LFC conducted experiments, acquired and analyzed data. LFC and SD designed the research studies and analyzed data. GB, LFC, and SD wrote the manuscript.
